# Availability and affordability of antimalarial and antibiotic medicines in Malawi

**DOI:** 10.1371/journal.pone.0175399

**Published:** 2017-04-18

**Authors:** Felix Khuluza, Lutz Heide

**Affiliations:** 1 Pharmacy Department, College of Medicine, University of Malawi, Blantyre, Malawi; 2 Pharmaceutical Institute, Eberhard Karls-Universität Tübingen, Tübingen, Germany; University of Groningen, NETHERLANDS

## Abstract

**Background:**

Availability and affordability of medicines are key determinants of universal health coverage, yet achieving them presents a major challenge especially in low-income countries. We here present an analysis of availability and prices of antimalarial and antibiotic medicines in public, faith-based and private health facilities in Malawi. Medicines are provided free of charge in the public health care system of Malawi. In contrast, facilities of the Christian Health Association of Malawi (CHAM) usually charge their patients for medicines, as do private for-profit facilities.

**Methods:**

As part of a study on medicine quality, samples of six antimalarial and six antibiotic medicines were collected in 31 health facilities in four districts of southern Malawi. These included 15 public facilities (i.e. health centres, district hospitals and central hospitals), eight CHAM and eight private facilities. Random selection was used in choosing the included health facilities. The availability of medicines was recorded, including the number of units which could be collected of each medicine, as well as the prices of medicines which were charged in CHAM and private facilities. These data were analyzed using the standard methodology developed by the World Health Organization (WHO) and Health Action International (HAI).

**Results:**

Availability of the antimalarials artemether/lumefantrine and sulfadoxine/pyrimethamine, which are provided with financial support from international donors, was high in public and CHAM facilities (93% and 100%, respectively). However, availability of antibiotics was much lower (e.g. 40% availability of amoxicillin tablets/capsules in public health centres). Medicine prices were lower than reported from many other countries. The median price ratio (MPR) to a wholesale international procurement price was 2.8 in CHAM facilities and even lower in the private sector (MPR 2.3). Nevertheless, for 10 of the 12 investigated medicines the cost for one course of treatment exceeded the daily wage of a low-paid government worker in Malawi and therefore had to be considered as unaffordable for a major part of the population.

**Conclusions:**

Continued efforts are required to improve the availability of essential medicines in Malawi. The free provision of medicines in the public health care system remains important in order to achieve universal health coverage, due to the low income in this country.

## Introduction

Medicines are an essential part of any health care system. Access to safe, effective, quality and affordable medicines has been included into the Sustainable Development Goals (SDGs) of the United Nations [1], as a key step towards achieving universal health coverage. Yet, in a survey in 25 low- and middle-income countries published in 2014, the median availability of essential medicines was found to be only 61.5% overall, and as low as 40% in the public sector [2]. The 67^th^ World Health Assembly in 2014 has urged member states to collect information on medicine shortages and on their causes, and to develop strategies to prevent and mitigate the associated problems [3]. We here report such information from Malawi.

Malawi is a low-income country in sub-Saharan Africa. In 2010, 71% of Malawi’s population lived below the international poverty line of 1.90 US $/day [4]. Approximately 60% of all health services are provided by the public health centres and hospitals, 37% by facilities of the Christian Health Association of Malawi (CHAM) and the rest by private for-profit health practitioners [5]. Essential health care, including essential medicines, is provided free of charge in all public health facilities. In contrast, CHAM facilities usually charge their patients for services and medicines [5], and so do private practitioners. Private health insurance covers only a small part of the urban population, while the majority of Malawi people have no access to complementary health insurance [5].

Medicine prices, availability and affordability can be analyzed with a standard methodology developed by WHO and Health Action International (HAI) [6]. This methodology has been used in multiple studies worldwide [2, 7–14]. Availability of medicines is also recorded within the Service Availability and Readiness Assessment (SARA) developed by WHO and USAID [15] which was applied in surveys in Malawi’s neighbor states Tanzania [16] and Zambia [17] but not yet in Malawi itself. Choi and Ametepi [18] critically compared different methods for the measurement of medicine availability. These authors reported that on-site investigation by study investigators consistently yields lower and more reliable availability estimates than reports prepared and sent by the health facility staff themselves. These authors also criticized the usual definition of availability as “availability of at least one non-expired unit of the respective medicine”, since it fails to provide information on whether the amount of medicine on-hand is sufficient to meet patient needs. Information also on the amount on-hand is therefore desirable.

Only few data have been published on the availability and affordability of medicines in Malawi. In 2007, a summary report from six countries on the availability and affordability of medicines for chronic diseases, using the WHO/HAI methodology, included data from the public and the private for-profit sectors in Malawi [13]. A study on prices and affordability of medicines of three asthma medicines, carried out by questionnaire in 52 countries [19], also included Malawi. Lufesi *et al*. [20] reported in 2007 on the availability of five antibiotics and three antimalarial medicines in eight public health centres in Malawi’s Lilongwe district. An earlier study [21] investigated the availability of the antimalarial sulfadoxine/pyrimethamine (SP) in Malawi’s Blantyre district.

We recently reported on the quality of medicines in southern Malawi, investigating samples of antimalarial and antibiotic medicines which were collected from 31 health facilities and drug outlets [22]. During the sample collection for that study, data were recorded on the availability and prices of the 12 investigated medicines. We now analyzed these data following the WHO/HAI methodology [6] in order to provide here a comprehensive report on the availability and affordability of antimalarial and antibiotic medicines in public and private health facilities in Malawi.

## Methods

### Medicine collection

Samples of 12 different antimalarial and antibiotic medicines were collected from 31 health facilities and drug outlets in southern Malawi with the primary aim to analyze medicine quality. The sampling strategy is explained in detail in the report on the quality analysis [22]. The study included the urban centre of Blantyre with its surrounding district, as well as three rural districts of southern Malawi. In each district, at least two public health centres were randomly selected, including facilities in remote rural locations. Medicines were collected from these public health centres as well as from the district hospitals of each district and from the central hospital in Blantyre. If CHAM health facilities or private drug outlets were situated in the vicinity of the public facilities, medicines were collected from these as well. In total, samples were collected from ten public health centres, four public district hospitals or district health offices, one public central hospital, eight CHAM facilities, two licensed pharmacies, three licensed drug stores and three non-licensed street vendors. The names of the investigated facilities are not reported in this study for reasons of confidentiality.

In order to allow for chemical analysis of medicine quality, it was attempted to collect samples of 150 tablets or capsules each of solid oral formulations, and 50 vials each of injectable formulations, if possible. If the full amount was not available at a given site, the amount available was recorded. In order not to cause drug stock-outs by our sample collection, a replacement for the sampled medicines was offered, using prepacked medicines carried by the investigators. In CHAM and private facilities, also the prices paid for the collected medicines were recorded.

Samples of the following six antimalarial and six antibiotic medicines were collected:

- artemether/lumefantrine 20 mg/120 mg tablets;
- sulfadoxine/pyrimethamine 500 mg/25 mg tablets;
- quinine hydrochloride inj. 300 mg/ml, 2ml vial;
- quinine sulfate 300 mg tablets;
- artesunate/amodiaquine 100 mg/270 mg tablets;
- dihydroartemisinin/piperaquine 40 mg/320 mg tablets;
- phenoxymethylpenicillin 250 mg tablets;
- amoxicillin 250 mg capsules/tablets;
- ciprofloxacin 250 (or 500) mg tablets;
- chloramphenicol 250 mg capsules;
- amoxicillin/clavulanic acid 500 mg/125 mg (or 250 mg/125 mg) tablets;
- cefuroxime (as axetil) 250 (or 500) mg tablets.

The Malawi Essential Medicines Lists (MEML) of 2009 and 2015 [23, 24] specify the level of health institution at which each medicine is normally permitted for use: only at central hospitals; or at both central and district hospitals; or on all levels of health care facilities including health centres. The above-mentioned 12 medicines were chosen in order to comprise examples from each of these three levels of use, as well as medicines which are not contained in the MEML and were therefore expected to be available only in the private sector (Table 1).

**Table 1:** Medicine availability in different types of health facilities in southern Malawi.

Availability according to MEML ^1^			Public Health Centres (n = 10)		Public District Hospitals (n = 4)		Public Central Hospital (n = 1)		Public Facilities overall (n = 15)		CHAM Facilities (n = 8)		Street Vendors (n = 3)		Private Drug Stores (n = 3)		Private Pharmacies (n = 2)		Private Facilities overall (n = 8)	
2009	2015																			
**H**	**H**	**Artemether/lumefantrine 20 mg/120 mg tbl**	9/10	**90%**	4/4	**100%**	1/1	**100%**	14/15	**93%**	8/8	**100%**	0/3	**0%**	2/3	**67%**	2/2	**100%**	4/8	**50%**
**H**	**H**	**Sulfadoxine/pyrimethamine 500 mg/25 mg tbl**	9/10	**90%**	4/4	**100%**	1/1	**100%**	14/15	**93%**	8/8	**100%**	2/3	**67%**	2/3	**67%**	2/2	**100%**	6/8	**75%**
**H**	**H**	**Quinine hydrochloride inj. 300 mg/ml, 2ml vial**	4/10	**40%**	4/4	**100%**	1/1	**100%**	9/15	**60%**	3/8	**38%**	0/3	**0%**	0/3	**0%**	1/2	**50%**	1/8	**13%**
**H**	**-**	**Phenoxymethylpenicillin 250 mg tbl**	0/10	**0%**	0/4	**0%**	0/1	**0%**	0/15	**0%**	5/8	**63%**	2/3	**67%**	1/3	**33%**	1/2	**50%**	4/8	**50%**
**D**	**H**	**Amoxicillin 250 mg cps/tbl**	4/10	**40%**	4/4	**100%**	1/1	**100%**	9/15	**60%**	6/8	**75%**	2/3	**67%**	1/3	**33%**	2/2	**100%**	5/8	**63%**
**D**	**D**	**Artesunate/Amodiaquine 100 mg/270 mg tbl**	0/10	**0%**	4/4	**100%**	1/1	**100%**	5/15	**33%**	0/8	**0%**	0/3	**0%**	0/3	**0%**	2/2	**100%**	2/8	**25%**
**D**	**D**	**Quinine sulfate 300 mg tbl**	1/10	**10%**	2/4	**50%**	0/1	**0%**	3/15	**20%**	6/8	**75%**	0/3	**0%**	1/3	**33%**	2/2	**100%**	3/8	**38%**
**C**	**D**	**Ciprofloxacin 250 (or 500) mg tbl**	4/10	**40%**	4/4	**100%**	1/1	**100%**	9/15	**60%**	8/8	**100%**	1/3	**33%**	1/3	**33%**	2/2	**100%**	4/8	**50%**
**C**	**C**	**Amoxicillin/clavulanic acid 500/125 (or 250/125) mg tbl**	0/10	**0%**	0/4	**0%**	0/1	**0%**	0/15	**0%**	1/8	**13%**	0/3	**0%**	0/3	**0%**	2/2	**100%**	2/8	**25%**
**N**	**-**	**Chloramphenicol 250 mg cps**	1/10	**10%**	2/4	**50%**	1/1	**100%**	4/15	**27%**	5/8	**63%**	0/3	**0%**	1/3	**33%**	2/2	**100%**	3/8	**38%**
**-**	**-**	**Dihydroartemisinin/pipera-quine 40 mg/320 mg tbl**	0/10	**0%**	0/4	**0%**	0/1	**0%**	0/15	**0%**	0/8	**0%**	0/3	**0%**	1/3	**33%**	2/2	**100%**	3/8	**38%**
**-**	**-**	**Cefuroxime (as axetil) 250 (or 500) mg tbl**	0/10	**0%**	0/4	**0%**	1/1	**100%**	1/15	**7%**	0/8	**0%**	1/3	**33%**	0/3	**0%**	2/2	**100%**	3/8	**38%**

^1^ The Malawi Essential Medicines Lists (MEML) of 2009 and 2015 specify the level of health institution at which the medicine is normally permitted for use: H = at health centre, district hospital and central hospital levels; D = at district hospital and central hospital levels only; C = at central hospital level only. N = level of use not specified.— = not included in MEML. In the present study, the availability of each medicine was nevertheless surveyed in each facility, and often differed from the availability foreseen in MEML.

If the indicated strength was not available at a given collection site, an alternative strength was sampled. This resulted in the collection of several samples of ciprofloxacin 500 mg tablets, amoxicillin/clavulanic acid 250/125 mg tablets and cefuroxime (as axetil) 500 mg tablets. The respective strength and the price were and recorded.

We speculated that especially brand medicines may be subject to falsification. Therefore, if medicines with generic and brand name were available, the brand name medicine was to be collected, and if several brand name medicines were available, the most expensive brand name medicine was to be sampled. It turned out, however, that in most sites only a single type of the respective medicine was available. Branded generic medicines were found to be no more expensive than generic medicines sold under their international non- proprietary names (see Results).

### Calculation of medicine availability

The calculation of medicine availability, prices and affordability was carried out according to the methodology published by WHO/HAI [6]. Medicine availability was defined as the percentage of facilities which had unexpired stock of the respective medicine at the time of the visit, irrespective of the amount and the strength available.

### Calculation of medicine prices

Medicines were obtained free of charge in all public health facilities, but were paid for in CHAM and in private facilities. Medicines were paid in local currency (Malawi Kwacha, MWK). Prices were converted to US $ using the exchange rate of April 2015 (1 US $ = 440.6675 MWK), the month in which most of the medicines were collected.

As suggested by the WHO/HAI manual [6], the ratio of the observed median prices to an international reference price was calculated, resulting in the median price ratio (MPR). The median supplier price from the MSH International Drug Price Indicator Guide of 2014 [25] was used as reference. If medicines of two different strengths were collected, median prices and MPRs were calculated separately for both strengths.

Medicines provided free of charge were not included in the calculation of median prices. In one CHAM facility, ciprofloxacin tablets were obtained at a price ten times lower than in all other facilities. It appears likely that this was an error of the accountant, and this price was not included in the calculations.

### Calculation of courses of treatment and of medicine affordability

The amount of tablets/capsules/vials required for one course of treatment was obtained from the Malawi Standard Treatment Guidelines [23, 24], from the WHO/HAI manual [6], from the WHO Model Formulary 2008 [26] and from the WHO ATC/DDD Index [27], using the dosage and treatment duration for the most common adult disease treated with the respective medicine, e.g. adult respiratory infection in the case of amoxicillin tablets/capsules. For amoxicillin tablets/capsules, different daily doses are given in different sources [6, 7, 24, 27], and we followed the WHO/HAI manual [6] in this case. For quinine injections, a treatment duration of three days (before changing to oral medication) was chosen. Prices of medicines collected in two different strengths were converted to reflect the preferred strength in our study.

As a measure of local affordability, the number of day's wages needed to purchase a course of treatment was calculated from the daily salary of the lowest-paid unskilled government worker in Malawi (551 MWK = 1.25 US $) [6, 28].

### Ethical approval

The study was approved by the College of Medicine Research and Ethics Committee, Blantyre, Malawi, under number P.05/14/1571, and by the national medicines regulatory authority (Pharmacy, Medicines and Poisons Board of Malawi).

## Results

### Availability of antimalarials and antibiotics

Table 1 summarizes the availability of medicines in different types of health facilities and drug outlets. In the 15 investigated public facilities, the availability of the antimalarials artemether/lumefantrine tablets and sulfadoxine/pyrimethamine tablets was 93%, and in the facilities of the Christian Health Association of Malawi (CHAM) even 100%. However, quinine injections for the treatment of severe malaria in adults were only available in 40% of public health centres and in 38% of the CHAM facilities (Table 1).

Artesunate/amodiaquine tablets, the second-line treatment for malaria in Malawi, were found in all public hospitals but not in any of the public health centres and neither in the CHAM facilities. Quinine sulfate tablets, intended to be used in combination with clindamycin for the treatment of uncomplicated malaria in the first trimester of pregnancy [24], were unavailable in two of the district hospitals, as well as in the central hospital. Dihydroartemisinin/piperaquine is included into the WHO guidelines for the treatment of malaria [29], but not into the Malawi Essential Medicines List. Accordingly, it was not found in any of the public health facilities, and neither in any of the CHAM facilities.

In 2015 the Malawi government published a new version of its essential medicines list [24], eliminating the use of phenoxymethylpenicillin tablets and replacing it with amoxicillin tablets/capsules. Correspondingly, phenoxymethylpenicillin was unavailable in all public health facilities, but still available in five of the eight CHAM facilities. Amoxicillin tablets/capsules were found to be available in 100% of the hospitals, but only in 75% of the CHAM facilities and in 40% of the public health centres.

The combination of amoxicillin with clavulanic acid is more expensive than many other antibiotics (see below), and the Malawi Essential Medicines List restricts its use to the central hospitals. However, at the time of our visit it was also unavailable in the central hospital, as well as in seven of the eight CHAM facilities.

Cefuroxime tablets are not contained in MEML [24] and neither in the WHO Essential Medicines List [30]. In our study, it was found in the central hospital but not in the other public or in the CHAM facilities.

The two licensed pharmacies included in our study were well stocked, having either ten or even all twelve of the investigated medicines in stock. In contrast, the licensed drug stores showed varying and often low availability of medicines. Of the three illegal street vendors investigated, one offered five of the twelve investigated medicines. The two other ones, however, offered only one or two of the medicines, respectively (S1 Table, Supporting Information).

S1 Table (Supporting Information) shows whether the full requested amount of each sample (150 tablets/capsules or 50 vials of each medicine) or only a partial amount could be collected in each site. In the public and CHAM facilities, the full amount could be collected in 87% of the cases, showing that medicines were usually available in quantities useful for treatment. In the private facilities, the full amount could only be collected in 48% of the cases, indicating that these facilities stock only smaller amount of medicines, probably for financial reasons.

**Fig 1:**
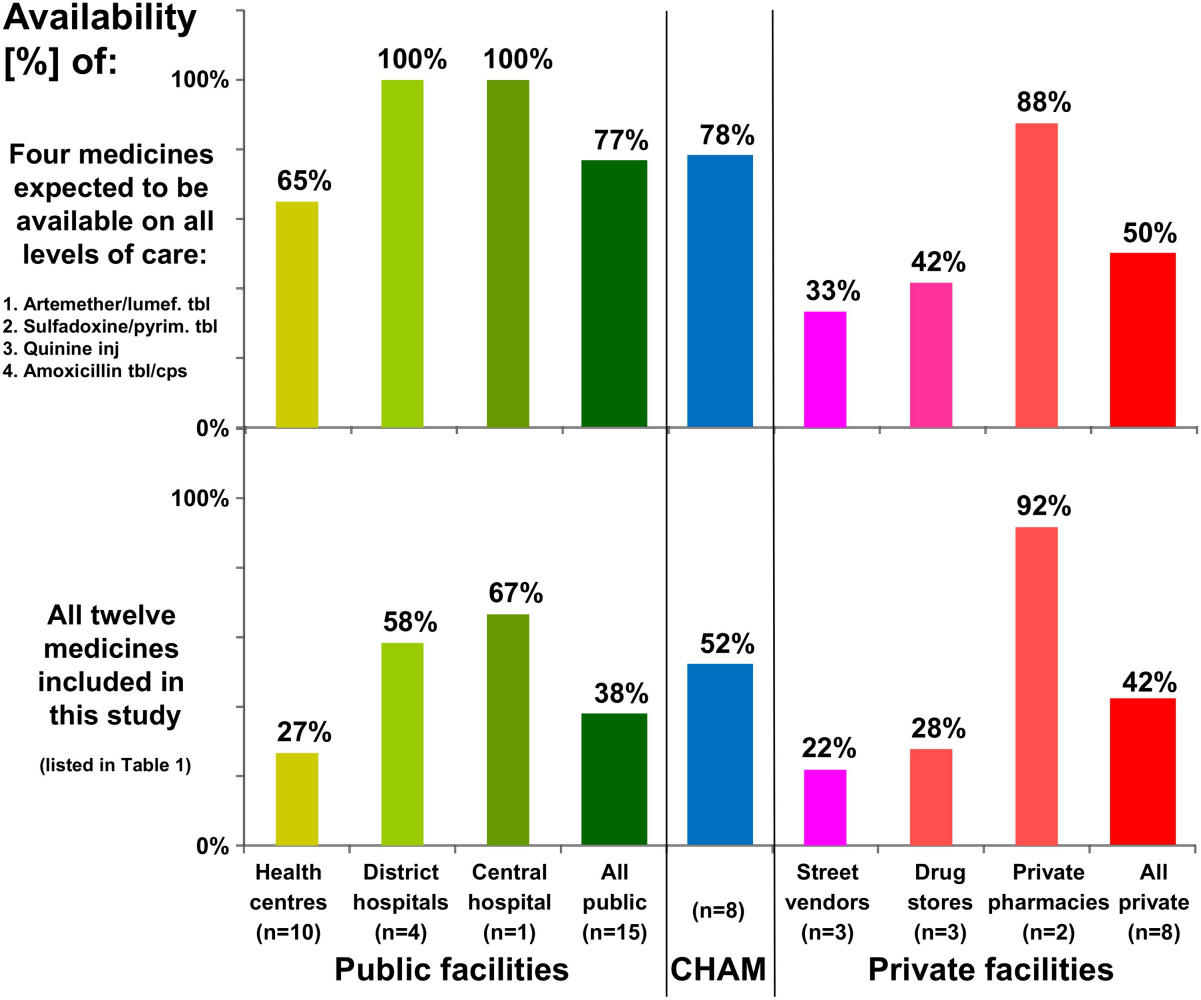
Availability of selected antibiotic and antimalarial medicines in Malawi. CHAM = Christian Health Association of Malawi.

As depicted in Fig 1, the four medicines which according to MEML should be available on all levels of the public health care system showed 100% availability in the public hospitals but only 65% in public health centres. Availability of all twelve medicines included in this study was found to be highest (92%) in private pharmacies. In faith-based (= CHAM) facilities, medicine availability was only slightly better than in public facilities (Fig 1).

### Medicine prices

Following the WHO/HAI methodology [6], median prices were calculated for all investigated medicines. As explained in the Methods section, the ratio of these median prices to the international reference price in the MSH International Drug Price Indicator Guide of 2014 [25] was calculated as median price ratio (MPR) (Table 2). Public health facilities in Malawi provide essential medicines free of charge,therefore Table 2 only shows prices of medicines collected in CHAM or private facilities. Few medicines are provided free of charge also in CHAM facilities. These include artemether/lumefantrine tablets which are provided under international donor programs, and sulfadoxine/pyrimethamine tablets provided for free in two of the investigated CHAM facilities, under service level agreements with the government for maternal and child health [5]. Medicines provided free of charge were not included into the median price calculations.

**Table 2:** Medicine prices (in US cent per tbl or vial) in different types of health facilities in southern Malawi.

Medicine	Strength	MSH Reference price per tbl/vial (US cent)	Christian Health Association of Malawi facilities (n = 8)										Private street vendors, drug stores and pharmacies (n = 8)									
			CHAM facility no.								Me-dian (US cent)	Me-dian price ratio	Street vendor no.			Drug store no.			Pharmacy no.		Me-dian (US cent)	Me-dian price ratio
			1	2	3	4	5	6	7	8			1	2	3	1	2	3	1	2		
**Phenoxymethyl-penicillin tbl**	250 mg	**1.7**	*3*.*4*		10.7			*9*.*1*	4.5	*5*.*7*	**5.7**	**3.3**		*3*.*4*	*6*.*8*	*4*.*3*				*4*.*1*	**4.2**	**2.4**
**Amoxicillin cps/tbl**	250 mg	**1.8**	3.4	4.9	10.7	11.3	6.8	11.3			**8.7**	**4.7**		6.8	6.8	*5*.*7*			*4*.*5*	*3*.*5*	**5.7**	**3.1**
**Amoxicillin/ clavulanic acid tbl**	500/125 mg	**20.0**	*45*.*4*								**45.4**	**2.3**								*31*.*9*	**31.9**	**1.6**
	250/125 mg	**13.5**																	*56*.*7*		**56.7**	**4.2**
**Ciprofloxacin tbl**	250 mg	**2.1**	4.5		*10*.*7*	*11*.*3*				*6*.*8*	**8.7**	**4.1**										
	500 mg	**4.3**		1.0^1^			*9*.*1*	*11*.*3*	*6*.*8*		**9.1**	**2.1**		*6*.*8*		*21*.*6*			*9*.*1*	*11*.*0*	**10.0**	**2.3**
**Chloramphenicol cps**	250 mg	**2.6**		8.8	9.1			*11*.*3*	4.5	*6*.*8*	**8.8**	**3.5**				6.0			*6*.*8*	*4*.*1*	**6.0**	**2.3**
**Cefuroxime (as axetil) tbl**	250 mg	**13.3**												*15*.*9*					*34*.*0*	*32*.*9*	**32.9**	**2.5**
**Artemether/ lumefantrine tbl**	20 mg/120 mg	**17.0**	0.0	0.0	0.0	*0*.*0*	*0*.*0*	*0*.*0*	*0*.*0*	*0*.*0*	**0.0**	**0.0**				*14*.*2*		*28*.*4*		*13*.*0*	**14.2**	**0.8**
	40 mg/240 mg	**n/a**																	*22*.*7*		**22.7**	**n/a**
**Artesunate/ amodiaquine tbl**	100 mg/270 mg	**25.0**																	*52*.*9*	*45*.*4*	**49.2**	**2.0**
**Dihydroartemisinin/ piperaquine tbl**	40 mg/320 mg	**40.1**																*44*.*1*	*50*.*4*	*39*.*6*	**44.1**	**1.1**
**Sulfadoxine/ pyrimethamine tbl**	500 mg/25 mg	**3.7**	18.2	6.8	*0*.*0*^1^	11.3	0.0^1^	18.2	5.7	7.7	**9.5**	**2.5**	*20*.*4*	*15*.*9*		*22*.*7*		*34*.*0*	*15*.*1*	*16*.*6*	**18.5**	**5.0**
**Quinine sulfate tbl**	300 mg	**6.4**	13.6	15.3	14.5	11.3		*9*.*1*	11.3		**12.5**	**2.0**					13.6		*11*.*3*	*11*.*1*	**11.3**	**1.8**
**Quinine hydrochloride inj**	300 mg/ml, 2ml	**24.0**	22.7				*68*.*1*	*90*.*8*			**68.1**	**2.8**								49.5	**49.5**	**2.1**
**Overall median price ratio to MSH reference price**^2^			**2.8**										**2.3**									

Prices of generic medicines sold under INN names are shown in normal print, prices of branded generic medicines and of originator brands in *italics*. Prices of medicine samples which were found not comply with pharmacopeial specifications [22] are underlined.

^1^ Not included in calculations, see Methods.

^2^ Prices of artemether/lumefantrine were not included in the calculation of the overall median price ratio for CHAM and private facilities.

As shown in Table 2, prices in the private sector (MPR 2.3) were even lower than in CHAM facilities (MPR 2.8). In many countries, medicine prices in the private sector are inflated by the high cost of originator brand medicines [7]. In our study, the only originator brand encountered was Coartem^®^ tablets, an artemether/lumefantrine (AL) combination provided by the Novartis company at a reduced price to low- and middle-income countries under an agreement with WHO and with funding from international donors. 42% of the medicines collected in this study represented so-called branded generics, i.e. generic medicines sold under brand names given by the manufacturers. True generic medicines, sold under the international non-proprietary names (INN), were most frequently found in the public sector (68% of samples) and in the CHAM facilities (58%), but in the private sector they only constituted 13% of the collected samples. In Table 2, prices of branded generics are shown in *italics*. Notably, for both branded and generic medicines the overall median price ratio observed in this study was the same, i.e.2.5.

Table 3 shows the costs for one course of adult treatment with the medicines included in this study. Since medicine prices in private and CHAM facilities were similar, median prices from all facilities were used for this calculation (see Methods).

**Table 3:** Prices per course of treatment with different antibiotics and antimalarials.

Medicine	Strength	Course of treatment	No. of units per treatment	Median price per tbl/vial (US cent)	Price per course of treatment (US $)
Phenoxymethyl-penicillin tbl	250 mg	8 tbl/day, 7 days	56	4.5	**2.54**
Amoxicillin cps/tbl	250 mg	6 tbl/day, 7 days	42	6.8	**2.86**
Amoxicillin/ clavulanic acid tbl	500/125 mg	3 tbl/day, 7 days	21	45.4	**9.53**
Ciprofloxacin tbl	250 mg	4 tbl/day, 7 days	28	5.5	**1.53**
Chloramphenicolcps	250 mg	8 cps/day, 5 days	40	6.8	**2.72**
Cefuroxime (as axetil) tbl	250 mg	2 tbl/day, 7 days	14	32.9	**4.61**
Artemether/ lumefantrine tbl	20 mg/ 120 mg	8 tbl/day, 3 days	24	13.6	**3.27**
Artesunate/ amodiaquine tbl	100 mg/ 270 mg	2 tbl/day, 3 days	6	49.2	**2.95**
Dihydroartemisinin/ piperaquine tbl	40 mg/ 320 mg	3 tbl/day, 3 days	9	44.1	**3.97**
Sulfadoxine/ pyrimethamine tbl	500 mg/ 25 mg	3 tbl/day, single dose	3	16.3	**0.49**
Quininesulfate tbl	300 mg	6 tbl/day, 7 days	42	11.3	**4.77**
Quinine hydro-chloride inj	300 mg/ml, 2ml	2 vials/day, 3 days	6	58.8	**3.53**

See Methods for calculation of data in this table.

### Affordability of medicines

**Fig 2:**
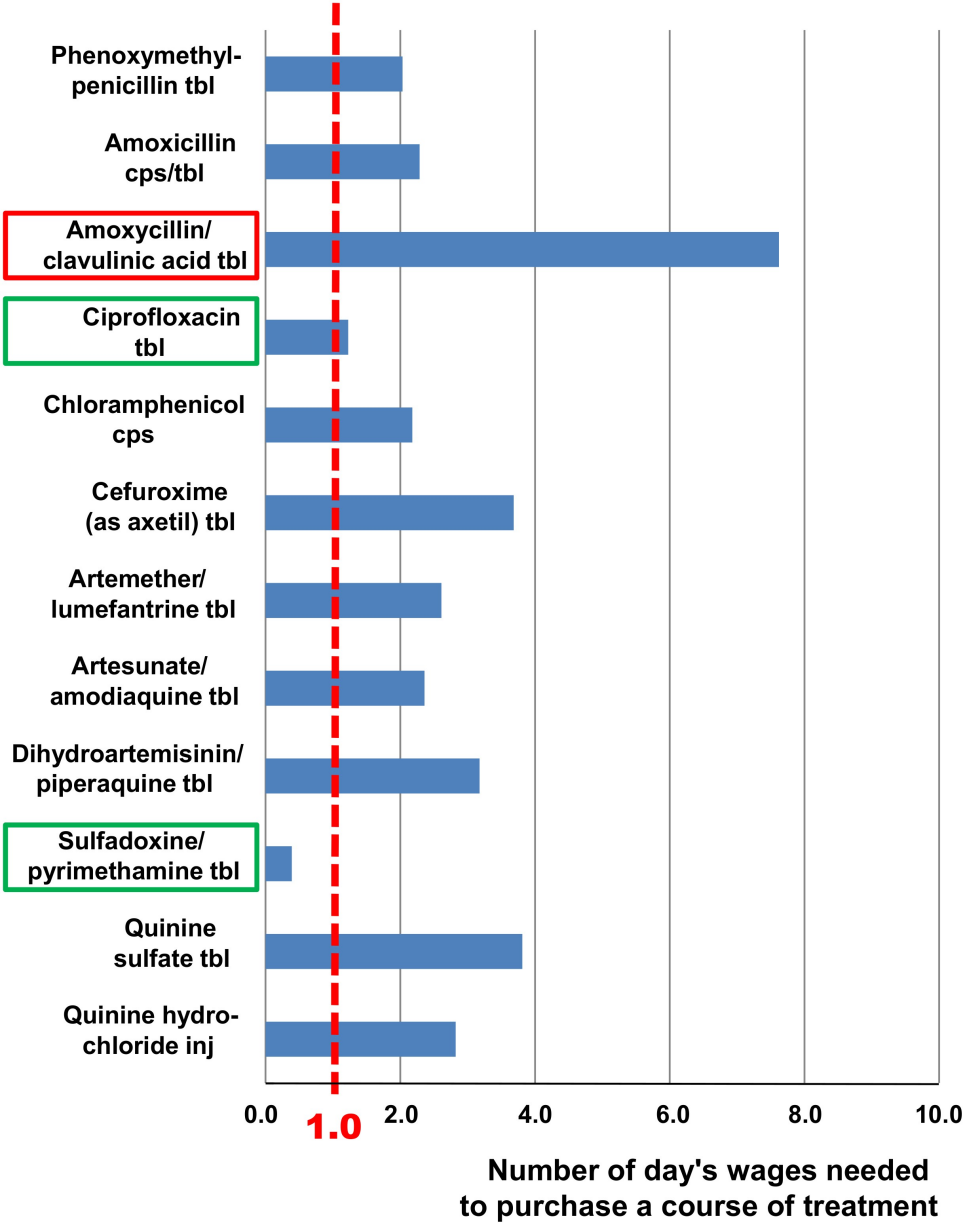
Affordability of antibiotic and antimalarial medicines in Malawi. The number of day's wages needed to purchase a course of treatment (see Table 3) was calculated from the daily salary of the lowest-paid unskilled government worker (1.25 US $). See Methods for calculation. The two most affordable medications are marked in green, and the most expensive one is marked in red.

Treatment affordability was estimated following the WHO/HAI methodology [6]. The daily salary of the lowest-paid unskilled government worker in Malawi, i.e. 1.25 US $ per day [28], was used to estimate the number of daily wages required for one course of treatment. The result is shown in Fig 2.

### Medicine quality and medicine prices

The collected samples were chemically analyzed for their quality [22]. This resulted in the detection of seven medicines which did not meet the specifications of the respective pharmacopeia. One of them (the sulfadoxine/pyrimethamine sample collected from street vendor 1; see Table 2) was falsified, and six were classified as substandard [22]. Notably, none of the samples collected in CHAM facilities, and only one of the samples collected in public health facilities, failed quality testing. Six out-of-specification medicines were found in the (licensed or non-licensed) private for-profit sector. The prices of these medicines are underlined in Table 2. Two of them were sold at prices below the median price charged in private facilities for this type of medicines, one of them at the median price, and three of them at a price above the median. Though the sample size is too small to draw definite conclusion, these results do not give evidence for a (positive or negative) correlation between medicine price and medicine quality within the samples investigated. The results show, however, that the prevalence of poor-quality medicines was higher in the (licensed or non-licensed) private for-profit sector than in public or faith-based health facilities [22].

## Discussion

### Availability of medicines

As shown in Table 1, availability of the first-line antimalarial artemether/lumefantrine tablets as well as availability of sulfadoxine/pyrimethamine tablets (intended for the intermittent preventive treatment of malaria in pregnancy [23, 24]) was high in public and CHAM facilities. These medicines are provided to the Malawi health care system through a donor-funded program involving the Global Fund, USAID and the UK Department of International Development. Our findings show the success of the Malawi government, the faith-based organizations and the donors in making these antimalarial medicines widely available in Malawi. In contrast, quinine injections which are life-saving for the treatment of severe malaria in adults showed quite low availability (40% in public health centres and 38% in CHAM facilities). For comparison, a survey in Kenya, Namibia, Rwanda, Tanzania and Uganda, including both public and NGO/faith-based facilities, showed on average 66% availability of artemether/lumefantrine tablets, 78% availability of sulfadoxine/pyrimethamine tablets, and 69% availability of quinine injections [18]. In Sierra Leone, availability of the first-line artemisinin-based combination therapy, i.e. artesunate/amodiaquine tablets, was 97% in public facilities, but availability of quinine injections was only 26% [9].

In Malawi, artesunate/amodiaquine is the second-line antimalarial medication and recommended by the Malawi Essential Medicines List to be available in the central and district hospitals but not in the health centres. Indeed, we found 100% availability on central and district hospital level and 0% on health centre level (Table 1). Therefore, availability of this medicine was exactly as planned. If, however, availability data would be aggregated into an overall “availability in the public sector”, this would result as only 33% (Table 1) and falsely suggest a very poor availability of this medicine. Many publications on medicine availability aggregate the availability data into the broad categories “public sector” and “private sector”, without further differentiation. Our data in Table 1 and Fig 1 show that important information may be obscured in such an aggregation of data. Also Robertson *et al*. [31] suggested that the limited stratification of health facilities in surveys using the WHO/HAI methodology [6] may limit the usefulness of the resulting data.

Amoxicillin tablets/capsules were found to be available only in 75% of the CHAM facilities and in 40% of the public health centres, indicating a serious gap in the availability of first-line antibacterial therapy. The situation in Tanzania was reported to be similar, with amoxicillin availabilities of 55% in the public sector and 66% in the faith-based facilities [16]. Zambia, on the other hand, has a reputation for a well-functioning medicines supply system, and amoxicillin availability was reported to be 92% and 97% in public and in faith-based facilities, respectively [17].

Amoxicillin/clavulanic acid was unavailable in the central hospital, as well as in seven of the eight CHAM facilities. This is a regrettable lack of a very safe and effective antibacterial treatment.

Ciprofloxacin is a potent broad-spectrum antibiotic, but the overuse of this and other gyrase inhibitors results in a rapid increase of resistance, causing worldwide concern [32]. MEML 2015 restricts its use to the district and central hospitals. However, contrary to these rules we found the medicine to be available in in four out of the ten public health centres, and also in all CHAM facilities. Likewise, chloramphenicol capsules which are not contained in MEML 2015 were available in many of the public and CHAM facilities. These findings are likely to indicate an overuse of these inexpensive antibiotics.

### Prices and affordability of medicines

As confirmed by several reports [5], essential medicines are provided free of formal or informal charges in all public health facilities in Malawi. Yet, due to geographic disparities in the accessibility of public health services, and due to shortages of medicines, of equipment and of staffing at the public facilities, the population frequently turns to CHAM facilities for health care. The latter facilities are also often believed to provide better service [5]. In contrast, the private for-profit sector is of limited importance for the provision of medicines and health services in rural areas of Malawi [5].

Overall, we found medicine prices in Malawi to be lower than reported from many other countries, as evidenced by an overall median price ratio (MPR) of 2.5. Interestingly, prices in the private sector (MPR 2.3) were even lower than in CHAM facilities (MPR 2.8) (Table 2). Possibly, CHAM facilities use some revenues from medicine sales to cover general operating expense, as has been noted in other low- and middle-income countries [7].

Gelders *et al*. [33] have suggested that patient prices should be considered as acceptable in private pharmacies if the MPR is ≤ 2.5. The MSH reference price [25] used in the calculation of the MPR is a price for bulk international procurement, usually not including freight and insurance. Therefore, the cited cut-off points by Gelders *et al*. [33] for acceptable prices are ambitious. A United Nations report of 2012 [34] states that in low- and middle-income countries, MPRs were on average 5.3 in the private sector, and 3.1 in the public sector. Even higher MPRs are reported in other studies [7, 8, 11]. Our study confirms, however, that the goal of an MPR ≤ 2.5 in the private sector is attainable.

According to the WHO/HAI methodology [6], medicine costs are considered as affordable when they amount to one day’s wages or less for one course of treatment. As shown in Fig 2, despite the favorable medicine prices most treatments are still unaffordable for a large part of Malawi’s population, due to the low income in this country. This finding is in agreement with the result of a recent study in Malawi, reporting that medical costs at CHAM and private facilities are perceived by the population as main barriers preventing access to health care [5].

### Limitations of this study

The sample collection within this study was carried out as part of a study onmedicine quality. Therefore, only a limited number of medicines and health facilities could be included. While data analysis followed the validated WHO/HAI methodology for availability and affordability studies [6], the sampling strategy deviated from that methodology in some aspects, following guidelines for medicine quality studies instead [35]. In accordance with WHO/HAI methodology, data collection for this study was carried out at a single point of time. As mentioned by Robertson *et al*. [31], it is desirable that single time point studies are complemented by a regular, continuous monitoring of medicine availability. This is currently attempted in Malawi by the establishment of a Logistics Management Information System (LMIS) within the Ministry of Health [36].

## Conclusions

Medicine availability in public and CHAM health facilities in Malawi was found to be high for the first-line antimalarials provided under international donor programs, evidencing the success of the governmental and non-governmental organizations involved in these efforts. However, availability was low for several of the investigated antibiotics, clearly falling behind the availability of such medicines in neighboring Zambia [17]. Continued efforts are required to improve the availability of such life-saving medications.

Medicine prices in Malawi were lower than reported from many other countries, especially in the private for-profit sector. Nevertheless, most treatments were still unaffordable for a large part of Malawi’s population, due to the low income in that country. The free provision of essential medicines in the public health systems remains important for achieving universal health coverage in Malawi.

## Supporting information

S1 Table Medicine availability in health facilities in southern Malawi. (PDF)
