# Supplementary material for: Availability and affordability of antimalarial and antibiotic medicines in Malawi
Source: PLoS One. 2017 Apr 18;12(4):e0175399. doi: 10.1371/journal.pone.0175399 (PMC5395150; doi:10.1371/journal.pone.0175399)
Supplement: S1 Table — (PDF) [file pone.0175399.s001.pdf]

Availability according to MEML [1] 2009	2015	Health Facility:	Public Health Centres (n=10)											Public District Hospitals (n=4)					Public Central Hospital (n=1)	
			Public HC 1	Public HC 2	Public HC 3	Public HC 4	Public HC 5	Public HC 6	Public HC 7	Public HC 8	Public HC 9	Public HC 10	**Total availibility**	Publich DH 1	Publich DH 2[3]	Publich DH 3	Publich DH 4[3]	**Total availibility**	Public CH 1	**Total availibility**
**2009**	**2015**	**District[2]:**	C	C	P	P	N	N	B	B	B	B		C	P	N	B		B	
**H**	**H**	**Artemether/lumefantrine 20 mg/120 mg tbl**	72	90	156	150	0	168	168	162	162	162	**9/10 (90 %)**	720	150	150	168	**4/4 (100%)**	168	**1/1 (100%)**
**H**	**H**	**Sulfadoxine/pyrimethamine 500 mg/25 mg tbl**	0	107	150	100	150	150	150	150	150	150	**9/10 (90%)**	150	150	150	150	**4/4 (100%)**	150	**1/1 (100%)**
**H**	**H**	**Quinine hydrochloride inj. 300 mg/ml, 2ml vial**	10	20	0	0	0	50	0	0	50	0	**4/10 (40%)**	50	50	50	50	**4/4 (100%)**	50	**1/1 (100%)**
**H**	**-**	**Phenoxymethylpenicillin 250 mg tbl**	0	0	0	0	0	0	0	0	0	0	**0/10 (0%)**	0	0	0	0	**0/4 (0%)**	0	**0/1 (0%)**
**D**	**H**	**Amoxicillin 250 mg cps/tbl**	111	0	150	0	0	150	150	0	0	0	**4/10 (40%)**	150	150	150	150	**4/4 (100%)**	150	**1/1 (100%)**
**D**	**D**	**Artesunate/Amodiaquine 100 mg/270 mg tbl**	0	0	0	0	0	0	0	0	0	0	**0/10 (0%)**	150	150	150	150	**4/4 (100%)**	150	**1/1 (100%)**
**D**	**D**	**Quinine sulfate 300 mg tbl**	0	0	0	0	0	150	0	0	0	0	**1/10 (10 %)**	0	150	0	150	**2/4 (50%)**	0	**0/1 (0%)**
**C**	**D**	**Ciprofloxacin 250 (or 500) mg tbl**	0	0	0	0	0	150	150	150	150	0	**4/10 (40%)**	100	150	150	150	**4/4 (100%)**	200	**1/1 (100%)**
**C**	**C**	**Amoxicillin/clavulinic acid 500/125 (or 250/125) mg tbl**	0	0	0	0	0	0	0	0	0	0	**0/10 (0%)**	0	0	0	0	**0/4 (0%)**	0	**0/1 (0%)**
**N**	**-**	**Chloramphenicol 250 mg cps**	0	97	0	0	0	0	0	0	0	0	**1/10 (10 %)**	150	0	150	0	**2/4 (50%)**	150	**1/1 (100%)**
**-**	**-**	**Dihydroartemisinin/piperaquine 40 mg/320 mg tbl**	0	0	0	0	0	0	0	0	0	0	**0/10 (0%)**	0	0	0	0	**0/4 (0%)**	0	**0/1 (0%)**
**-**	**-**	**Cefuroxime (as axetil) 250 (or 500) mg tbl**	0	0	0	0	0	0	0	0	0	0	**0/10 (0%)**	0	0	0	0	**0/4 (0%)**	150	**1/1 (100%)**

Health Facility:	Christian Health Association of Malawi Facilities (n=8)									Private Drug Stores (n=3)				Private Pharmacies (n=2)			Illegal Street Vendors (n=3)			
	CHAM 1	CHAM 2	CHAM 3	CHAM 4	CHAM 5	CHAM 6	CHAM 7	CHAM 8	**Total availibility**	Drug Store 1	Drug Store 2	Drug Store 3	**Total availibility**	Pharmacy 1	Pharmacy 2	**Total availibility**	Street vendor 1	Street vendor 2	Street vendor 3	**Total availibility**
**District[2]:**	C	P	P	P	N	N	B	B		C	P	N		B	B		C	C	B	
**Artemether/lumefantrine 20 mg/120 mg tbl**	120	162	162	168	162	144	162	168	**8/8 (100%)**	96	0	96	**2/3 (67%)**	96	54	**2/2 (100%)**	0	0	0	**0/3 (0%)**
**Sulfadoxine/pyrimethamine 500 mg/25 mg tbl**	100	150	150	150	150	150	150	150	**8/8 (100%)**	90	0	150	**2/3 (67%)**	150	111	**2/2 (100%)**	81	120	0	**2/3 (67%)**
**Quinine hydrochloride inj. 300 mg/ml, 2ml vial**	30	0	0	0	50	50	0	0	**3/8 (38%)**	0	0	0	**0/3 (0%)**	0	11	**1/2 (50%)**	0	0	0	**0/3 (0%)**
**Phenoxymethylpenicillin 250 mg tbl**	150	0	150	0	0	150	150	150	**5/8 (63%)**	80	0	0	**1/3 (33%)**	0	150	**1/2 (50%)**	0	100	150	**2/3 (67%)**
**Amoxicillin 250 mg cps/tbl**	150	160	150	150	150	150	0	0	**6/8 (75%)**	80	0	0	**1/3 (33%)**	150	150	**2/2 (100%)**	0	50	150	**2/3 (67%)**
**Artesunate/Amodiaquine 100 mg/270 mg tbl**	0	0	0	0	0	0	0	0	**0/8 (0%)**	0	0	0	**0/3 (0%)**	30	12	**2/2 (100%)**	0	0	0	**0/3 (0%)**
**Quinine sulfate 300 mg tbl**	100	150	150	150	0	150	150	0	**6/8 (75%)**	0	150	0	**1/3 (33%)**	150	150	**2/2 (100%)**	0	0	0	**0/3 (0%)**
**Ciprofloxacin 250 (or 500) mg tbl**	100	150	150	150	150	150	150	150	**8/8 (100%)**	80	0	0	**1/3 (33%)**	150	150	**2/2 (100%)**	0	150	0	**1/3 (33%)**
**Amoxicillin/clavulinic acid 500/125 (or 250/125) mg tbl**	80	0	0	0	0	0	0	0	**1/8 (13%)**	0	0	0	**0/3 (0%)**	150	80	**2/2 (100%)**	0	0	0	**0/3 (0%)**
**Chloramphenicol 250 mg cps**	0	150	107	0	0	150	150	150	**5/8 (63%)**	150	0	0	**1/3 (33%)**	150	150	**2/2 (100%)**	0	0	0	**0/3 (0%)**
**Dihydroartemisinin/piperaquine 40 mg/320 mg tbl**	0	0	0	0	0	0	0	0	**0/8 (0%)**	0	0	81	**1/3 (33%)**	153	153	**2/2 (100%)**	0	0	0	**0/3 (0%)**
**Cefuroxime (as axetil) 250 (or 500) mg tbl**	0	0	0	0	0	0	0	0	**0/8 (0%)**	0	0	0	**0/3 (0%)**	70	40	**2/2 (100%)**	0	80	0	**1/3 (33%)**

S1 **Table: Medicine availability in health facilities in southern Malawi.**

For each medicine and facility, the number of tabl/cps/vials which were collected during this study is given. If available, approximately 150 tbl/cps or 50 vials were collected. Lower numbers indicate that the full amount was not available. Total availability is calculated for each type of facility, without consideration whether the full amount or only a smaller amount was available.

[1] The Malawi Essential Medicines Lists (MEML) of 2009 and 2015 specify the level of health institution at which the medicine would normally be permitted for use: H = at Health Centre, District Hospital and Central Hospital levels; D = at District Hospital and Central Hospital levels only; C = at Central Hospital level only. N = level of use not specifed; - = not included in MELM.

[2] Districts: C = Chikwawa, P = Phalombe, N = Nsanje, B = Blantyre

[3] Phalombe and Blantyre district do not have district hospitals. Data are therefore from the District Health Offices of these two districts.
